# Abstracts

**DOI:** 10.1155/S1463924683000322

**Published:** 1983


### Abstracts

### Page 124

### A microcomputer-based injection system for investigating the influence of atmospheric pressure on chromatographic response in the analysis of gases

B. R. Wybrow and E. J. Greenhow

A sample injection system has been developed for investigating injection technique in the gas chromatographic analysis of gases, with particular emphasis on control of the technique in order to measure the influence of atmospheric pressure on chromatographic response.

The injection system is driven by a microcomputer-based sequence controller, which utilizes specially written software and specially built electronic interfacing and pressure measuring equipment.

Automatic injection of a sample of gas can be effected at any chosen pressure between vacuum and 25 $lpf/in^2$ into a three-column chromatographic system which uses 'external to the column oven' flow-switching techniques to separate automatically the gases $H_2$, $O_2$, $N_2$, CO, $CH_4$, $CO_2$, $C_2H_4$, and $C_2H_6$.

The functions of the control lines of the microcomputer are summarized, electronic equipment is described, and the way in which column switching and back-flushing times in the chromatographic analysis have been automatically optimized is briefly explained.

The work shows quantitatively the extent to which variations in atmospheric pressure can influence response when analysing nitrogen using a concentration sensitive detector with helium carrier gas.

Use of the standard 'stopped-flow' technique of injection is shown to minimize the effect of variations in atmospheric pressure on response because the effects of changes in atmospheric pressure at the sample loop and detector on chromatographic response are almost mutually compensatory.

Predictions are made concerning the influence of variations in atmospheric pressure on response: (1) for a solely mass-sensitive detector which could suffer significant changes in response, and (2) for a mass-sensitive flame ionization detector which could suffer additionally from the influence of atmospheric pressure on ionization efficiency in a manner dependent on the chromatographic conditions and gases analysed.

### Un système d'injection basé sur microordinateur pour l'étude de l'influence de la pression atmosphérique sur la réponse chromatographique dans l'analyse des gaz

B. R. Wybrow and E. J. Greenhow

Un système d'injection d'échantillons a été développé pour l'étude de la technique d'injection dans l'analyse par chromatographie en phase gazeuse de gaz avec emphase particulière sur le contrôle de la technique de façon à mesurer l'influence de la pression atmosphérique sur la résponse chromatographique.

La séquence du système d'injection est contrôlée par un microordinateur qui utilise un logiciel spécial et également une interface électronique conçue spécialement et du matériel pour mesurer la pression. L'injection automatique d'un échantillon de gaz peut être effectuée à n'importe quelle pression entre le vide et 25 psi dans un système chromatographique à 3 colonnes qui utilise une technique de 'switching' de flux extérieur au four de colonnes qui sépare automatiquement les gaz $H_2$, $O_2$, $N_2$, CO, $CH_4$, $CO_2$, $C_2H_4$ et $C_2H_6$.

Les fonctions de contrôle du microordinateur sont brièvement présentées, le matériel électronique est décrit et la façon dans laquelle le changement des colonnes et le temps de rinçage dans l'analyse chromatographique ont automatiquement été optimés, sont brièvement expliqués.

Ce travail montre quantitativement l'influence des variations atmosphériques sur la réponse lors de l'analyse de l'azote utilisant un détecteur sensible à la concentration avec l'hélium comme gaz-porteur.

L'utilisation des techniques standard 'stopped flow' d'injection rend minimal l'effet de variations de la pression atmosphérique sur la réponse car l'effet de changements de pression atmosphérique sur la vanne d'injection et le détecteur se compensent mutuellement.

Des prédictions sont faites concernant l'influence de variations de la pression atmosphérique sur la réponse (1) pour des détecteurs uniquement sensibles à la masse qui risquent de souffrir de changement significatifs dans la réponse et (2) pour un détecteur sensible à la masse par ionisation en flamme qui, en plus, risque de souffrir de l'influence de la pression atmosphérique sur l'efficacité de ionisation d'une façon dépendant des conditions chromatographiques et des gaz à analyser.

### Ein mikrocomputergesteuertes Injektionssystem für die Untersuchung des Luftdruckeinflusses auf das Chromatographiesignal bei der Analyse von Gasen

B. R. Wybrow and E. J. Greenhow

Für die Untersuchung der Injektionstechnik bei der gaschromatographischen Analyse von Gasen wurde ein Probeninjektionssystem entwickelt. Die Steuerung der Technik wurde speziell in Hinblick auf die Erfassung des Einflusses des Luftdrucks auf das chromatographische Signal entworfen.

Das Einspritzsystem wird von einem Mikrocomputer-Taktgeber gesteuert, welcher speziell zu diesem Zweck verwendete Software und speziell konstruierte elektronische Interface- und Druckmessgeräte verwendet.

Die automatische Injektion einer Gasprobe kann bei irgendeinem Druck zwischen Vakuum und 25 psi durchgeführt werden. Die Einspritzung erfolgt in einen Dreikolonnenchromatographen, der ausserhalb der Kolonnenheizung liegende Durchflussumschalter verwendet, um automatisch die Gase $H_2$, $O_2$, $N_2$, CO, $CH_4$, $CO_2$, $C_2H_4$, und $C_2H_6$ zu trennen.

Die Funktionen der Steuerleitungen des Mikrocomputers sind angegeben, die elektronischen Geräte werden beschrieben und die Art, wie die Kolonnenumschaltung und die Spülzeiten in der chromatographischen Analyse automatisch optimiert wurden, wird kurz erklärt.

Die Arbeit zeigt quantitativ das Ausmass, in welchem Schwankungen des Atmosphärendrucks das Signal beeinflussen, wenn Stickstoff mit Helium als Trägergas und unter Verwendung eines konzentrationsempfindlichen Detektors analysiert wird.

Es wird gezeigt, dass die Verwendung der Standard 'stopped-flow' Einspritztechnik die Wirkung der Einflüsse des atmosphärischen Drucks auf das Signal minimalisiert, weil die Wirkungen dieser Aenderungen des atmosphärischen Drucks auf die Probenschlaufe und den Detektor, und damit auf das Chromatographiesignal sich gegenseitig fast kompensieren.

Für den Einfluss der Schwankungen des Atmosphärendrucks auf das Signal eines nur massenempfindlichen Detektors wird eine Prognose gemacht. Diese zeigt, dass ein solcher Detektor signifikante Aenderungen des Signals erfahren könnte. Eine Prognose wird auch gemacht für den massenempfindlichen Flammenionisationsdetektor, der zusätzlich einen Einfluss der atmosphärischen Schwankungen auf die Ionisationseffizienz erfährt, der von den chromatographischen

Bedingungen und vom analysierten Gas abhängt.

---

### Page 136

### Use of pattern-recognition display techniques to visualize the data contained in complex data-bases. A case study*

M. P. Derde *et al.*

The object of the paper is to illustrate that display and related pattern-recognition techniques permit investigation of multivariate data-sets. This was demonstrated with a practical example: the authentification of the geographical origin of wine. The data-set consisted of 195 different French wines, each was analysed for its amino-acid pattern. The information included in the data was visualized using display methods (principal components analysis—non-linear mapping), as well as supervised methods (linear discriminant analysis) and unsupervised methods (hierarchical clustering).

On the basis of the displays obtained it could be concluded that the differentiation of wines according to the origin must be possible by using amino-acid patterns.

### L'utilisation de méthodes de reconnaissance des formes pour visualisation de données multivariées complexes. Example pratique

M. P. Derde *et al.*

Le but de cet article est d'illustrer que des méthodes de visualisation et des méthodes de reconnaissance des formes permettent d'étudier des données multivariées. Ceci est démontré à l'aide d'un exemple pratique, notamment la recherche de l'origine géographique d'un échantillon de vin. Les données consistaient en des concentrations en acides aminés présents dans 195 vins d'origine connue. L'information contenue dans ces données a été visualisée à l'aide de l'analyse des composantes principales et de méthodes similaires comme la non linear mapping, ainsi que par des méthodes de classification (analyse discriminante linéaire et classification hiérarchique).

On conclut que la discrimination des vins est possible par l'application de ces méthodes.

---

### Page 146

### The measurement of serum paracetamol using a discrete analyser

R. S. Campbell *et al.*

The adaptation of a commercially available kit for the measurement of serum paracetamol to a discrete analyser is described. The manual method is a two-stage reaction involving the specific enzymatic degradation of paracetamol and the colorimetric determination of the reaction product, p-aminophenol. The limitations imposed by such a two-stage reaction are discussed and experiments to minimize these limitations described.

Results are presented for reagent stability, final colour stability, linearity, carry-over, within-batch and between-batch precision and accuracy.

From these results it would appear that the enzymatic paracetamol assay can be adapted to any discrete analyser that may be used in the emergency laboratory.

### La détermination du paracétamol de sérum utilisant un analyseur discret

R. S. Campbell *et al.*

L'adaptation d'un set de mesure commercial pour le dosage du paracétamol de serum à un analyseur discret est décrite. La méthode manuelle est une réaction en deux étapes utilisant la dégradation spécifique enzymatique du paracétamol et la détermination colorimétrique du produit de réaction p-aminophénol. Les limitations imposées par une telle réaction à deux étapes sont discutées et des expériences qui permettent de minimaliser ces limitations sont décrites.

Des résultats sont présentés pour la stabilité du réactif, la stabilité de la couleur finale, la linéarité, la contamination et la précision et reproductibilité entre échantillons et entre séries. Ces résultats montrent que le dosage enzymatique du paracétamol peut être adapté à n'importe quel analyseur discret utilisé dans le laboratoire d'urgences.

### Die Bestimmung von Serum-Paracetamol mit einem diskontinuierlichen Analysator

R. S. Campbell *et al.*

Es wird die Anpassung eines kommerziell erhältlichen Reagenzsatzes für die Bestimmung von Serum-Paracetamol an einem diskontinuierlichen Analysator beschrieben. Die manuelle Methode besteht aus einer zweistufigen Reaktion, die den spezifischen enzymatischen Abbau von Paracetamol und die kolorimetrische Bestimmung des Reaktionsprodukts, Paraaminophenol, umfasst. Die Limitierungen einer solchen zweistufigen Reaktion werden diskutiert, und es werden Experimente beschrieben, die diese Limitierungen minimalisieren.

Resultate werden angeführt für die Reagenzienstabilität, Stabilität der Farbe, Linearität, Probenverschleppung, Präzision innerhalb einer Serie und von Serie zu Serie und Genauigkeit.

Diesen Resultaten scheint man entnehmen zu können, dass die enzymatische Paracetamol- Analyse auf jeden diskontinuierlichen Analysator angepasst werden kann, der in einem Notfall Labor Verwendung finden kann.

---

### Page 150

### Evaluation of two kinetic methods for serum amylase using a Cobas Bio centrifugal analyser

M. G. Anderson and A. M. Kelly

Two kinetic methods for the measurement of serum amylase were compared with the Phadebas end-point method. Both kinetic methods (Boehringer and Beckman) were adapted for use on a Cobas Bio centrifugal analyser resulting in between-batch CVs of 3–6%. The methods were rapid, convenient and inexpensive compared with the Phadebas method.

### Evaluation de deux méthodes cinétiques pour l'amylase de sérum utilisant un analyseur centrifuge Cobas Bio

M. G. Anderson and A. M. Kelly

Deux méthodes cinétiques pour la mesure de l'amylase de sérum ont été comparées avec la méthode à point final Phadebas. Les deux méthodes cinétiques (Boehringer et Beckman) ont été adaptées pour l'utilisation sur un analyseur centrifuge Cobas Bio résultant en un coéfficient de variation entre séries de 3 à 6%. Les méthodes étaient rapides, pratiques et peu chères comparées avec la méthode Phadebas.

### Evaluation zweier kinetischer Methoden für die Bestimmung von Serum-Amylase auf dem Cobas Bio Zentrifugal-Analysator

M. G. Anderson and A. M. Kelly

Die beiden kinetischen Methoden für die Bestimmung von Serum-Amylase werden mit der Phadebas-Endpunkt-Methode verglichen. Beide kinetischen Methoden (Boehringer und Beckman) wurden an die Verwendung auf einem Cobas Bio Zentrifugal-Analysator angepasst und ergaben Variationskoeffizienten von 3–6%. Die Methoden sind im Vergleich mit der Phadebas-Methode schnell, bequem und billig.

### Page 153

### Plasma albumin determination on the Cobas Bio centrifugal analyser using bromocresol green

J. R. Cowie and G. O. Evans

A method is fully described for the determination of plasma albumin using two reagents, and it is based on the binding of albumin to bromocresol green in the presence of citrate buffer. The dye-binding method is compared to an immunoturbidimetric method using the same centrifugal analyser; good agreement between methods is obtained for the range 22–57 g/l. The performance of the method in external quality-assessment schemes is also described.

### Détermination de l'albumine de plasma sur l'analyseur centrifuge Cobas Bio utilisant le vert Bromocrésole

J. R. Cowie and G. O. Evans

Une méthode pour la détermination de l'albumine de plasma est décrite utilisant deux réactifs. Cette méthode se base sur la fixation de l'albumine sur le vert de bromocrésol dans la présence d'un tampon de citrate. Cette méthode par fixation de colorant est comparée à une méthode immunoturbidimétrique utilisant le même analyseur centrifuge; une bonne corrélation entre les méthodes est obtenue pour la plage 22 à 57 g/l. La performance de la méthode pour le contrôle de qualité externe est également décrite.

### Die Bestimmung von Plasma-Albumin mit Bromkresolgrün auf dem Biozentrifugalanalysator Cobas

J. R. Cowie and G. O. Evans

Es wird eine Methode für die Bestimmung von Plasma-Albumin, die zwei Reagenzien benützt, vollständig beschrieben. Sie basiert auf der Bindung des Albumins an Bromkresolgrün in der Gegenwart von Zitratbuffer. Die Farbstoffbindungsmethode wird mit einer immunoturbidimetrischen Methode, die den gleichen Zentrifugalanalysator benützt, verglichen, im Bereich von 22–57 g/l wird gute Uebereinstimmung der Resultate der beiden Methoden erhalten. Die Leistungsfähigkeit der Methode in externen Qualitätsbeurteilungs-Schemata wird ebenfalls beschrieben.

---

### Page 155

### The quality control of measured data*

L. Leisztner and P. Barna

In two-dimensional analytical measurements analytical information is always biased and of varied standard deviation because of the noise. A method for estimating the standard deviation of random errors of measured signal samples is described. The method was tested by simulating 'noisy' chromatographic measurements on a computer. The generated and the estimated standard deviation of the random errors did not show any significant difference.

---

### Page 157

### Analytical performances achieved by the 'Aria II' automatic system in an inter-laboratory survey for triiodothyronine and thyroxine assays

A. Pilo *et al.*

The analytical performances of the automatic Aria II system for $T_3$ and $T_4$ assays has been evaluated from data collected during a one-year inter-laboratory survey involving over 100 laboratories; about 10% of participants used the Aria II. The accuracy of the Aria II measurements was estimated in respect of both consensus means and calibrated samples. $T_3$ results were affected by a positive bias, which ranged from 17% to 7% in the low and high samples (0·8–3·2 ng/ml) respectively, while $T_4$ measurements exhibited only a slight bias (3% to −3% for concentrations ranging from 4 to 16 μg/100 ml). The between-laboratory and between-batch precisions, computed from results of hidden replicates, were 12·4 CV% for $T_3$ and 9·2 CV% for $T_4$. These findings, when compared with figures obtained from six manual methods used by some participants in the survey, indicate that the Aria II system assays $T_3$ and $T_4$ with a relatively good precision.

### Performances analytiques obtenues par le système automatique Aria II dans une étude interlaboratoire pour l'analyse de la triiodothyronine et de la thyroxine

A. Pilo *et al.*

La performance analytique du système automatique Aria II pour l'analyse $T_3$ et $T_4$ a été étudiée sur la base de données collectionnées pendant une année et couvrant plus de 100 laboratoires; plus de 10% des participants utilisaient le système Aria II. L'exactitude des mesures avec Aria II a été étudiée avec des échantillons calibrés et des moyennes de standard. Les résultats $T_3$ étaient trop hauts de 7 à 17% pour les valeurs faibles et hautes (0·8 à 3·2 ng/ml) tandis que les mesures $T_4$ variaient uniquement de +3% à −3% pour les concentrations entre 4 et 16 μg/ml. La précision entre laboratoires et entre séries donne un coéfficient de variation (CV) de 12·4% pour $T_3$ et 9·2% pour $T_4$. Ces données comparées avec des résultats obtenus par six méthodes manuelles utilisées par certains participants dans cette recherche, indiquent que l'analyse de $T_3$ et $T_4$ par Aria II donne une précision relativement bonne.

### Die analytische Leistung des Aria II Automaten in einer Untersuchung der Triiodothyronine- und Thyroxine-Analyse in vielen Laboratorien

A. Pilo *et al.*

Die analytische Leistung des automatischen Aria II Systems für $T_3$-$T_4$ Analysen wurde aufgrund von Daten, die während eines Jahres in mehr als 100 Laboratorien gesammelt wurden, evaluiert. Etwa 10% der Teilnehmer an dieser Untersuchung benützen den Aria II. Die Genauigkeit der Aria II Messungen wurde aufgrund der Consensus-Mittelwerte und von Resultaten kalibrierter Proben geschätzt. $T_3$ Resultate zeigten eine positive Abweichung, welche 17% bis 7% für tiefe respektive hohe Probenwerte (0,8 – 3,2 ng/ml) betrug. $T_4$ Messungen zeigten nur eine schwache Abweichung (3% bis – 3% für Konzentrationen von 4 bis 16 μg/100 ml). Die erreichte Präzision für Resultate verschiedener Laboratorien und Resultate verschiedener Serien, berechnet auf der Basis nicht erkennbarer Proben von bekannter Konzentration, betrug 12,4 CV% für $T_3$ und 9,2 CV% für $T_4$. Diese Zahlen, wenn sie mit dem Zahlenmaterial verglichen werden, die von verschiedenen Versuchsteilnehmern mit sechs manuellen Methoden erhalten wurden, zeigen, dass das Aria II System $T_3$ und $T_4$ Proben mit relativ guter Präzision analysiert.

---

* The publisher regrets that three translations (M. P. Derde *et al.*'s German abstract, and L. Leisztner and P. Barna's French and German abstracts) cannot be published until Vol. 5, No. 4.

